# Apelin-13 as a Potential Biomarker in Critical Illness

**DOI:** 10.3390/jcm12144801

**Published:** 2023-07-20

**Authors:** Marin Gergics, Gréta Pham-Dobor, Csilla Kurdi, Gergely Montskó, Krisztina Mihályi, Gábor Bánfai, Péter Kanizsai, Tamás Kőszegi, Emese Mezősi, László Bajnok

**Affiliations:** 1 1st Department of Internal Medicine, Medical School, University of Pécs, 7624 Pécs, Hungary; gergics.marin@gmail.com (M.G.); pham-dobor.greta@pte.hu (G.P.-D.); mezosi.emese@pte.hu (E.M.); 2 János Szentágothai Research Centre, University of Pécs, 7624 Pécs, Hungary; kurdi.csilla@pte.hu (C.K.); gergely.montsko@gmail.com (G.M.); koszegi.tamas@pte.hu (T.K.); 3 Department of Laboratory Medicine, Medical School, University of Pécs, 7624 Pécs, Hungary; 4 Department of Emergency Medicine, Medical School, University of Pécs, 7624 Pécs, Hungary; mihalyi.krisztina@pte.hu (K.M.); banfai.gabor@pte.hu (G.B.); kanizsai.peter@pte.hu (P.K.)

**Keywords:** copeptin, apelin-13, free cortisol, CRH, critical illness

## Abstract

Background: The adrenocortical system and copeptin as prognostic markers were intensively investigated in critical illness. The potential predictive power of apelin-13 as a biomarker is largely unknown. We aimed to investigate the prognostic role of apelin-13 in relation to free cortisol, aldosterone, CRH, and copeptin in critically ill patients. Methods: In this prospective observational study, 124 critically ill patients (64 men, 60 women, median age: 70 (59–78) years) were consecutively enrolled at the time of admission. All routinely available clinical and laboratory parameters were evaluated and correlated to hormonal changes. Results: Serum apelin-13 was 1161 (617–2967) pg/mL in non-survivors vs. 2477 (800–3531) pg/mL in survivors (*p* = 0.054). The concentrations of apelin-13 and CRH had strong positive correlations (r = 0.685, *p* < 0.001) and were significantly higher in surviving non-septic patients (Apelin-13 (pg/mL): 2286 (790–3330) vs. 818 (574–2732) *p* < 0.05; CRH (pg/mL) 201 (84–317) vs. 89 (74–233) *p* < 0.05). Apelin-13 and free cortisol were independent determinants of survival in the multivariate Cox regression analysis, while copeptin, CRH, or aldosterone were not. Conclusions: Beyond free cortisol, serum apelin-13 may also help refine prognostic predictions in the early phase of critical illness, especially in non-septic patients.

## 1. Introduction

### 1.1. Background

The mortality of patients requiring critical care is high and partly unpredictable. Complex prognostic scores have been created to improve the prediction of outcomes. However, despite intensive efforts, the predictive power of prognostic biomarkers is not optimal. Looking for new biomarkers would be essential not just to improve the prognostic role but for a better understanding of the pathomechanisms of critical conditions as well [1,2].

The prognostic roles of cortisol and arginine vasopressin (AVP) have been intensively studied in the literature.

Neuro-hormonal responses, including elevating cortisol levels, play a vital role in stress. Free cortisol concentration is typically high in critically ill patients due to the early activation of the hypothalamus-pituitary–adrenocortical axis (HPA) and decreased cortisol metabolism [3,4]. Previously, we demonstrated that free cortisol is an independent predictor of 30-day mortality in a mixed population of critically ill patients admitted to the ICU [5]. In addition to its prognostic value, free cortisol was reported to be a good marker of inflammatory response in septic shock [6].

Arginine vasopressin, or anti-diuretic hormone (ADH), is released from the posterior pituitary by increasing plasma osmolality and upon various stressors, such as hypovolemia, hypoxia, acidosis, and severe infections. The copeptin level is a reliable surrogate parameter for the AVP effect in healthy and critically ill patients [7]. Copeptin is elevated in patients admitted to the ICU, e.g., in a septic state [7,8,9]. Moreover, copeptin also serves as a prognostic marker; at admission, copeptin serum concentration is higher in non-survivors [10,11], and it is closely associated with severity scores (SAPS II and APACHE II) [12]. Copeptin is an independent predictor of survival in septic shock [7,13] and acute heart failure [14]. The apelinergic system is closely related to AVP and other stress reactions, but its potential role as a prognostic biomarker is largely unknown.

Apelin, a 36-aminoacid peptide hormone, is particularly abundant in the supraoptic and paraventricular nuclei, co-localizing with AVP in magnocellular neurons [15,16]. Apelin blocks the AVP secretion and action on the distal convoluted and collecting tubules of the kidney [17]. Its receptor has been detected in the cerebral cortex, hypothalamus, hippocampus, and pituitary gland [15]. Furthermore, the apelinergic system can be found in peripheral tissues: in the heart [18], gastrointestinal tract, skeletal muscle, liver, ovary, kidney, adipose tissue, lung, and endothelial cells [19].

In animal models, apelin’s in vivo effects on HPA function have been mediated by CRH and AVP-dependent mechanisms [20]. Apelin also regulates cardiovascular homeostasis, which is essential in regulating blood pressure, increasing cardiac output, and having a cardioprotective effect against oxidative stress [18,19,21]. Moreover, apelin is an endothelium-dependent vasodilator via the renin-angiotensin system (RAS) [17,22]. In addition to its essential role in cardiovascular homeostasis, apelin is involved in fluid homeostasis, glucose metabolism, and other physiological activities [17,21].

Apelin-13 has been identified as a significant apelin isoform in human plasma [23].

Corticotropin-releasing hormone (CRH) is a central regulator of the hormonal stress response, stimulating both corticotropin (ACTH) and AVP secretion [24]. The parvocellular CRH neurons also co-express AVP, which acts as a second ‘releasing factor’ for ACTH and CRH [25]. In contrast to ACTH, CRH is rarely measured in peripheral blood samples because it does not correlate well with those in the hypothalamic-hypophysial portal plasma. CRH is expressed in the brain, immune cells, and the gut, where gene expression is upregulated by lipopolysaccharide (LPS) [26]. To the best of our knowledge, there is no information about the serum CRH level at admission in critically ill patients.

### 1.2. Objectives

Our present study aimed to investigate a more complex interplay of the hypothalamic and adrenocortical systems in a mixed population of patients with critical illness. Based on the abovementioned data, the following hormones were selected for analysis as biomarkers: apelin-13, copeptin, CRH, free cortisol, and aldosterone.

## 2. Materials and Methods

### 2.1. Study Design, Setting

A prospective cohort study was carried out on critically ill patients admitted to the Intensive Care Unit of either Department of Emergency Medicine or 1st Department of Medicine, Clinical Center, the University of Pécs. Recruitment was achieved in two periods, between June and October 2012 and May 2019 and June 2020. Vital signs, clinical status, and routine laboratory parameters were monitored. The treatment of patients was thoroughly evaluated, and blood samples disturbed by glucocorticoid treatment were excluded from further analysis. The severity of the diseases was scored according to the SAPS II and the APACHE II scoring systems.

### 2.2. Participants

It was a mixed population of patients with medical emergencies. The patients were consecutively enrolled in two periods requiring intensive care due to vital organ dysfunction [27].

Patients with COVID-19, surgical procedure, or trauma were excluded. None of the patients received etomidate, ketoconazole, or any other drug influencing steroid metabolism. Eighteen patients needed complete cardiopulmonary resuscitation, and eight were defibrillated before admission.

Our study was performed by the ethical guidelines of the 2003 Declaration of Helsinki, and we obtained the permission of the Regional Research Ethical Committee of the University of Pécs. Written informed consent was obtained from the participants or the participants’ parent/legal guardian/next of kin to participate in the study.

### 2.3. Determination of Routine Laboratory Tests and Neurohormonal Mediators

At the time of admission, the chemistry panel and fully automated blood picture tests were determined using the standard laboratory diagnostic kits and automated instrumentation of the Department of Laboratory Medicine, University of Pécs (accreditation number: NAH-1-1553/2016).

At admission, blood samples were taken to measure the free cortisol, apelin-13, copeptin, CRH, and aldosterone levels. They were collected in plastic tubes in an anticoagulant-free Vacutainer (Becton Dickinson, Hungary Kft., Környe, Hungary). After centrifuging the collected blood samples at 2200× g for 10 min, serum was separated into aliquots in Eppendorf tubes and frozen under −80 °C. Sample preparation and the measurements for free cortisol analysis were performed according to the validated method using high-performance liquid chromatography coupled with high-resolution ESI-TOF mass spectrometry described by Montsko et al. [28].

Serum apelin-13, copeptin, and CRH levels were measured with the ELISA method using Human Apelin-13 ELISA kit (Catalog No.: abx252028, Abbexa Ltd., Cambridge, UK; intra-assay: CV < 10%, inter-assay: CV < 10%), Human Copeptin (CT-proAVP) ELISA kit (Catalog No.: abx252269, Abbexa Ltd., Cambridge, UK; intra-assay: CV < 10%, inter-assay: CV < 10%), and Human Corticotropin-Releasing Hormone (CRH) ELISA Kit (Catalog No.: MBS264947, MyBioSource; intra-assay: CV ≤ 8%, inter-assay: CV ≤ 12%) according to the manufacturer’s instructions on a BioTek Synergy HT plate reader at 450 nm. Serum aldosterone was measured using the radioimmunoassay method (Ref: IM1664, RIA-mat 280, Stratec).

### 2.4. Statistical Methods

Statistical analyses were carried out using SPSS 22.0 software. The Shapiro-Wilk test was used to check normal distribution; data are presented as mean ± SD in parameters with normal distribution, while median and interquartile values are in the non-normal distribution. To determine the relationship between parameters, Spearman’s correlation was used. Comparisons of two subgroups were made with Mann-Whitney U tests. The backward selection was used for binary logistic regression analyses, and a *p*-value of <0.1 was deemed significant. A *p*-value of <0.05 was regarded as significant, except for multiple logistic regression analyses; if linear methods with backward selection were used, a *p*-value of <0.1 was determined as significant. The beta value reflects the direction and the power of association. Cox regression analysis with backward selection was used to describe survival time; a *p*-value of <0.1 was significant.

## 3. Results

A total of 124 patients were recruited for the study. Patients represented a mixed population with a demand for critical care.

The key parameters of the patient population are shown in Table 1.

SAPS II score and hormonal parameters according to 30-day survival are shown in Table 2. Compared to the normal range, the free cortisol, CRH, and copeptin levels were highly elevated in both surviving and non-surviving patients with critical illnesses (no information regarding the normal range of apelin-13 is available.) The median sodium, potassium, creatinine, and urea values were within the normal limit.

The median and interquartile of the investigated hormonal parameters are separately demonstrated according to the main diagnosis of admission in Table 3.

Correlations of hormonal parameters with hormonal levels, 30-day mortality, severity score, and clinical parameters are demonstrated in Table 4.

Fifty-five patients (44%) had hypotension at admission. Significant correlations were found between hypotension and free cortisol (0.328, *p* < 0.001), copeptin (0.226, *p* = 0.012), aldosterone (0.221, 0 = 0.014), CRH (0.274, *p* = 0.002), and SAPS II (0.291, *p* = 0.001), but not with apelin-13 or 30-day mortality.

While investigating determinants of serum apelin-13 level by multiple linear regression analysis in two different models, CRH, SAPS II, serum sodium, potassium, and the presence of kidney injury were independent predictors (Table 5 and Table 6). Apelin-13 level was significantly elevated in patients with kidney failure (without vs. with kidney injury: 1439 (648–3250) vs. 2967 (1757–3836), *p* = 0.005). Using the same model, the independent predictors of serum CRH level were apelin-13 level and kidney injury (Table 7).

Hormone levels below and above the median of the SAPS II score are shown in Table 8.

Hormone levels according to the septic state are shown in Table 9. Free cortisol showed the sharpest difference of the investigated hormones: septic patients had almost six-fold elevation compared to the non-septic population. The CRH and copeptin were also highly elevated. The median of apelin-13 was higher in the septic group than the non-septic group, but due to the high interindividual variability and low number of septic patients, it was not statistically significant. The kidney function was affected by sepsis, and the values of both urea (*p* = 0.001) and creatinine (*p* = 0.005) were significantly elevated.

SAPS II score and hormonal parameters according to 30-day survival in the non-septic subgroup (N = 94 patients; 76% of the total population) are shown in Table 10.

### Cox Regression Analysis

Free cortisol and apelin-13 were significant independent predictors of mortality of the investigated hormonal parameters. In the whole population, free cortisol was the strongest predictor, while in the non-septic subgroup, apelin-13 became a stronger predictor of mortality than free cortisol (Table 11.). Survival function at the mean of apelin-13 and CRH in the whole population and according to the presence of sepsis is demonstrated in Supplementary Figure S1. No difference in the survival of those patients, whose apelin-13 and CRH levels were below and above the mean, was found except in the septic patient group where the apelin-13 level was significantly higher in non-survivors. Interestingly, an opposite trend was found in non-septic patients.

## 4. Discussion

Our current study investigated various hypothalamic and adrenal hormones in a population with a mixed critical illness. According to previous works, the free cortisol and copeptin serum concentrations were significantly higher among non-survivors [5,10,11]. Apelin-13 showed an opposite change, with a significantly decreased level in more severe cases represented above the median SAPS II score. The apelin-13 serum level was significantly lower in the subgroups of higher SAPS II severity scores and non-septic non-survivors. Furthermore, apelin-13 concentration significantly negatively correlated with the SAPS II severity score during univariate and multivariate analyses. Cox regression analysis was used to find the independent predictors of survival. Several models containing the routinely investigated laboratory parameters were used. Based on the sequence of removal, these models are appropriate to determine the importance of the individual factors. Of the investigated humoral parameters, apelin-13, in addition to free cortisol, was an independent determinant of survival. At the same time, copeptin, CRH, and aldosterone were dropped out. However, it is important to mention that the survival curves according to the mean of apelin-13 showed an opposite tendency in septic and non-septic patients. In the septic subgroup, highly elevated apelin-13 was related to poorer survival. In the non-septic group, a poorer survival trend was found by Cox regression analysis in those who had lower apelin-13 levels. Therefore, the regulation of apelin-13 in septic and non-septic critically ill conditions may be different and further studies are required to recruit a higher number of septic patients.

Apelin has been found to be an essential biomarker for heart failure [29]. Moreover, plasma apelin concentrations were depressed early after myocardial infarction, but did not correlate with the left ventricular function parameters [30]. In patients with ST-segment elevation myocardial infarction, the major adverse cardiovascular events were significantly more common in the low apelin group than in the high apelin group [31]. However, to the best of our knowledge, ours is the first study demonstrating a negative association between serum apelin-13 concentrations and the severity of critical illness. Contrary to our findings, Lesur et al. found no evidence of correlations between apelin-12, another apelin isoform, and either severity or outcome in critically ill patients exhibiting systemic inflammatory response syndrome [32]. These discrepant results may be due to the different patient populations (septic or non-septic; for example after myocardial infarction) and/or the different investigated apelin isoforms. For example, in the study of Lesur et al., even copeptin was not significantly higher in critically ill patients than in normal volunteers, despite the numerous concordant well-demonstrated results showing marked elevation of copeptin in these patients [7,8,9,12,33,34].

The biological efficacy of the apelin system is compromised under some environmental pressure. For example, in human sepsis, endogenous apelinergic levels rise early, and specific enzymatic breakdown activities potentially threaten endogenous apelin system reactivity and negatively impact the outcome [35]. Furthermore, the short-term exogenous apelin-13 infusion helps stabilize cardiorenal functions in ovine septic shock; however, this ability might be impaired by specific enzymatic systems triggered during the early course of human sepsis [35].

To the best of our knowledge, this is also the first study to investigate serum CRH concentrations in a critically ill population. Compared to the reference range, serum CRH was highly elevated in these patients, especially in septic ones, and positively correlated with apelin-13 and copeptin; however, interestingly, not with free cortisol. Furthermore, serum CRH has strongly determined apelin-13 levels among these patients. Moreover, similar to the apelin-13, serum CRH was significantly higher in surviving non-septic patients than in those deceased within 30 days. No previous human data are available regarding the strong correlation between CRH and apelin-13, so the explanation is only hypothetical; extreme stress reaction may be responsible for the elevation of both hormones. Our knowledge about the degradation of CRH and apelin-13 is also incomplete. It is well known that the elevation of cortisol levels in critical illness is partly due to the decreased degradation process [4]. It may explain the lack of correlation between CRH and free cortisol. Contrary to apelin-13 levels, CRH was not a significant parameter in the Cox regression analysis of survival.

Although both apelin-13 and CRH are basically expressed in the hypothalamus, their serum concentrations are determined by other sources and have weak relationships with their local effects on the central nervous system [21]. More complex regulation and stimulating factors can be presumed in these hormonal systems.

It has been previously demonstrated that the stress response of early admitted ICU patients is different in septic vs. non-septic conditions [32]. The septic subgroup of our patients had a more severe illness with a higher SAPS II score. Moreover, the level of the investigated hormones, except aldosterone, was higher among them than in non-septic patients, although the difference in the case of apelin-13 did not reach significance.

Serum aldosterone correlated to the free cortisol and SAPS II severity score; otherwise, aldosterone did not show any remarkable connections with the other parameters.

Many attempts have been made to improve survival in critical illness and to determine a more accurate prognostic score. There is a lack of convincing disease-modifying therapy in multi-organ failure. The investigation of the role of apelin-13 may have three benefits: to better understand the pathophysiological process and the complex regulation of this hormonal system, to find a prognostic marker that may be simpler than the currently used prognostic scores (which contains seventeen parameters), and to identify a potential therapeutic target.

The apelin system is obviously modified by critical illness, and these changes seem to be different in septic and non-septic conditions. In sepsis, the apelin-13 is extremely high; survival is better in those patients who have below the mean apelin-13 level, therefore, in this patient population, the elevation of apelin-13 may be a marker of severity, and a further increase of apelin-13 by the administration of exogenous apelin-13 is questionable. In the non-septic patient population, only apelin-13 remained a significant determinant of survival in multivariate Cox regression analysis. In non-septic patients with circulatory failure and low apelin-13 levels, exogenous apelin-13 administration might improve the prognosis.

### Limitations

There may be some possible limitations of the study. The sample size of the investigated population was relatively limited. This may explain why the serum apelin-13 elevation did not reach significance in the patients that survived compared to the non-survived subgroup of the whole population. However, the study’s limited size does not seem to influence our basic observations. Furthermore, no normal controls were included. However, this might not affect the observations related to the potential prognostic roles of the evaluated hormonal parameters at admission to the ICU. Moreover, other relevant humoral factors with potential significance, e.g., ACTH, could have also been tested that might have revealed other relevant hormonal interrelations.

## 5. Conclusions

Serum apelin-13 showed a decreased level in more severe cases of the mixed population with a critical illness. It appears that there is a considerable difference between the septic and non-septic populations with respect to apelin-13 levels. Moreover, the type of vital organ dysfunction markedly influenced not just the apelin-13 but all the other investigated hormones. The concentrations of apelin-13 and CRH had a strong positive correlation and both hormone levels were significantly higher in surviving non-septic patients. In the multivariate Cox regression analysis of the investigated hormonal parameters, apelin-13 and free cortisol were independent determinants of survival, while copeptin, CRH, or aldosterone were not.

## Figures and Tables

**Table 1:** Patient’s main characteristics.

Age (years, median, interquartile)	70 (59–78)
Gender (male/female)	64/60
30-day mortality rate	43 of 124 35%
Mechanical ventilation	46%
Catecholamine treatment	58%
Acute hemodialysis	27%
APACHE II score (median, interquartiles)	22 (17–29)
SAPS II score (median, interquartiles)	40 (32–60)
Diagnosis	
Sepsis	30
Acute heart failure	21
Pulmonary embolism	8
Acute myocardial infarction	8
Primary respiratory failure	20
Critical arrhythmias	10
Others	27

**Table 2:** SAPS II score and hormonal parameters as median (Q1-Q3), according to 30-day survival in the total population.

	Total Population	Normal Range	Survived on Day 30 (n = 81; 65%)	Deceased within 30 Days (n = 43; 35%)
**SAPS II** score ***	40 (32–60)	not applicable	**36** **(24–46)**	**60** **(42–70)**
**Free cortisol** (nmol/L) **	35 (10–126)	1–8	**25** **(5–89)**	**65** **(29–199)**
**Copeptin** (pg/mL) *	697 (459–1106)	4–52	**642** **(416–1175)**	**765** **(568–1055)**
Apelin-13 (pg/mL)	2024 (704–3320)	no data	2477 (800–3531)	1161 (617–2967)
CRH (pg/mL)	176 (80–356)	4–11	205 (85–357)	105 (75–322)
Aldosterone (pg/mL)	162 (79–354)	67–335	156 (76–296)	212 (93–419)

* *p*-value is between 0.05 and 0.01, ** *p*-value is between 0.01 and 0.001, *** *p*-value is below 0.001, bold shows significant parameters.

**Table 3:** The concentrations of hormonal parameters as median (Q1–Q3) differentiated by the underlying reasons for admission.

	Free Cortisol (nmol/L)	Apelin-13 (pg/mL)	CRH (pg/mL)	Aldosterone (pg/mL)	Copeptin (pg/mL)
Sepsis (n = 30)	117 (26–222)	2869 (1155–3766)	376 (107–801)	127 (69–395)	1566 (693–3379)
Acute heart failure (n = 21)	32 (5–150)	3107 (1254–5180)	235 (85–287)	197 (113–413)	880 (487–1479)
Pulmonary embolism (n = 8)	4 (3–8)	3037 (2503–3505)	336 (209–819)	117 (61–231)	405 (297–443)
Acute myocardial infarction (n = 8)	64 (4–117)	3434 (2825–5816)	318 (184–806)	173 (105–802)	664 (273–1632)
Primary respiratory failure (n = 20)	31 (12–72)	648 (489–2517)	89 (58–231)	227 (107–350)	652 (509–733)
Critical arrhythmias (n = 10)	26 (4–93)	2269 (823–3677)	158 (121–299)	114 (65–204)	831 (440–1385)
Others (n = 27)	36 (21–67)	732 (512–1742)	83 (70–204)	212 (67–386)	513 (449–745)

**Table 4:** Correlations of hormonal and severity parameters.

	Free Cortisol	Copeptin	Apelin-13	Aldosterone	CRH	SAPS II	30-Day Mortality
**Free cortisol**		0.217 *	−0.105	0.359 ***	0.098	0.480 ***	0.280 **
**Copeptin**	0.217 *		0.214 *	0.060	0.251 **	0.106	0.178 *
**Apelin**-**13**	−0.105	0.214 *		0.006	0.685 ***	−0.231 **	−0.173
**Aldosterone**	0.359 ***	0.060	0.006		0.028	0.197 *	0.101
**CRH**	0.098	0.251 **	0.685 ***	0.028		−0.079	−0.124
**SAPS** II	0.480 ***	0.106	−0.231 **	0.197 *	−0.079		0.510 ***
**30-day mortality**	0.280 **	0.178 *	−0.173	0.101	−0.124	0.510 ***	

* *p*-value is between 0.05 and 0.01, ** *p*-value is between 0.01 and 0.001, *** *p*-value is below 0.001, bold shows significant parameters.

**Table 5:** Determinants of serum apelin-13 level by multiple linear regression analysis.

Dependent variable: Apelin-13	
**Investigated Parameters**	**Beta-Value**
CRH ***	**0.405**
SAPS II *	**−0.197**
Sodium *	**−0.152**
Potassium *	**−0.196**
Age *	**0.160**
Free cortisol	−0.025
Copeptin	0.122
Aldosterone	−0.050
Creatinine	0.092
Urea	0.190
Sex	0.016
Sepsis	−0.122
R-squared	0.334
Adjusted R-squared	0.262

* *p*-value is between 0.1 and 0.01, *** *p*-value is below 0.001, bold shows significant parameters.

**Table 6:** Determinants of serum apelin-13 level by multiple linear regression analysis.

Dependent variable: Apelin-13	
**Investigated Parameters**	**Beta-Value**
CRH ***	**0.330**
SAPS II *	**−0.281**
Sodium *	**−0.142**
Age *	**0.211**
Kidney injury *	**0.263**
Potassium	−0.157
Free cortisol	−0.060
Copeptin	0.064
Aldosterone	−0.021
Creatinine	0.048
Urea	0.034
Sex	0.028
R-squared	0.361
Adjusted R-squared	0.292

* *p*-value is between 0.1 and 0.01, *** *p*-value is below 0.001, bold shows significant parameters.

**Table 7:** Determinants of serum CRH level by multiple linear regression analysis.

Dependent Variable: CRH	
**Investigated Parameters**	**Beta-Value**
Apelin-13 ***	**0.374**
Kidney injury *	**0.209**
SAPS II	−0.033
Sodium	0.086
Potassium	0.032
Age	0.018
Free cortisol	0.127
Copeptin	0.111
Aldosterone	0.028
Creatinine	−0.187
Urea	0.011
Sex	−0.094
R−squared	0.277
Adjusted R−squared	0.199

* *p*-value is between 0.1 and 0.01, *** *p*-value is below 0.001, bold shows significant parameters.

**Table 8:** Comparison of medians (Q1-Q3) of hormone levels below and above the median of the SAPS II score.

	Below the Median of SAPS II (n = 62)	Above the Median of SAPS II (n = 62)
Free cortisol (nmol/L) ***	**13 (3–60)**	**73 (31–202)**
Copeptin (pg/mL)	663 (434–1028)	714 (503–1740)
Apelin-13 (pg/mL) *	**2878 (854–3489)**	**1261 (618–3153)**
CRH (pg/mL)	204 (87–317)	127 (75–380)
Aldosterone (pg/mL)	131 (69–283)	215 (110–404)

* *p*-value is between 0.05 and 0.01, *** *p*-value is below 0.001, bold shows significant parameters.

**Table 9:** Comparison of medians (Q1-Q3) of SAPS II score and hormone levels between the septic and non-septic groups.

	No Sepsis (n = 94)	Sepsis (n = 30)
SAPS II score *	**39 (26–58)**	**49 (37–65)**
Free cortisol (nmol/L) **	**31 (6–93)**	**172 (38–277)**
Copeptin (pg/mL) ***	**650 (439–909)**	**1637 (694–2934)**
Apelin-13 (pg/mL)	1479 (645–3250)	3230 (2229–4013)
CRH (pg/mL) **	**132 (75–279)**	**573 (322–877)**
Aldosterone (pg/mL)	173 (82–341)	128 (67–544)

* *p*-value is between 0.05 and 0.01, ** *p*-value is between 0.01 and 0.001, *** *p*-value is below 0.001, bold shows significant parameters.

**Table 10:** SAPS II score and hormonal parameters as median (Q1-Q3), according to 30-day survival in the non-septic subgroup.

	Survived at Day 30 (n = 64)	Deceased within 30 Days (n = 30)
SAPS II score ***	**35 (23–43)**	**61 (41–69)**
Free cortisol (nmol/L) *	**24 (4–72)**	**36 (21–138)**
Copeptin (pg/mL) *	**542 (414–880)**	**749 (512–890)**
Apelin-13 (pg/mL) *	**2286 (790–3330)**	**818 (574–2732)**
CRH (pg/mL) *	**201 (84–317)**	**89 (74–233)**
Aldosterone (pg/mL)	158 (78–297)	224 (108–415)

* *p*-value is between 0.05 and 0.01, *** *p*-value is below 0.001, bold shows significant parameters.

**Table 11:** Multivariate Cox regression analysis of overall survival according to hormonal parameters.

Investigated Parameters	Chi-Square in the Whole Population	Chi-Square in the Nonseptic Group
Free cortisol	4.69 *	4.08
Apelin-13	3.33 *	3.20 *
Copeptin	0.01	0.70
CRH	0.09	0.02
Aldosterone	0.79	1.23
Number of observations	124	94

* *p*-value is between 0.1 and 0.01, bold shows significant parameters.

## Data Availability

The data supporting this study’s findings are available from the corresponding author, László Bajnok, upon reasonable request.

## Supplementary Materials

The following supporting information can be downloaded at: https://www.mdpi.com/article/10.3390/jcm12144801/s1, Supplementary Figure S1. Survival function at the mean of Apelin-13 and CRH in the whole population and according to the presence of sepsis.
