# Supplementary material for: Apelin-13 as a Potential Biomarker in Critical Illness
Source: J Clin Med. 2023 Jul 20;12(14):4801. doi: 10.3390/jcm12144801 (PMC10381233; doi:10.3390/jcm12144801)
Supplement: Supplementary file 1 [file jcm-12-04801-s001.zip › jcm-2450148-supplementary.pdf]

Supplementary Figure S1. Survival function at the mean of Apelin-13 and CRH in the whole population and according to the presence of sepsis

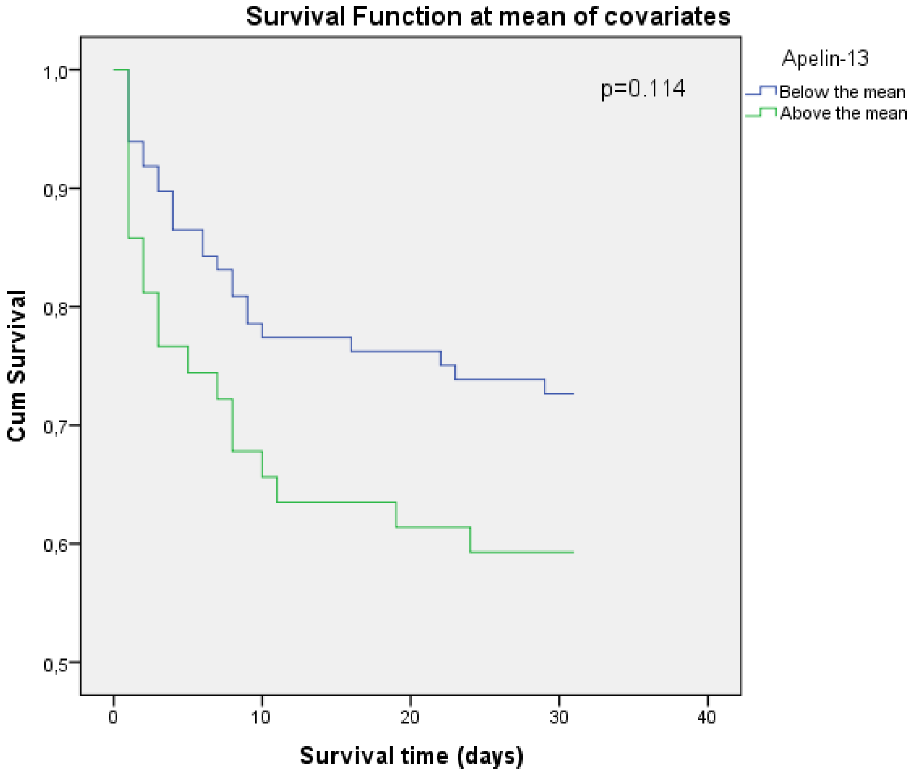

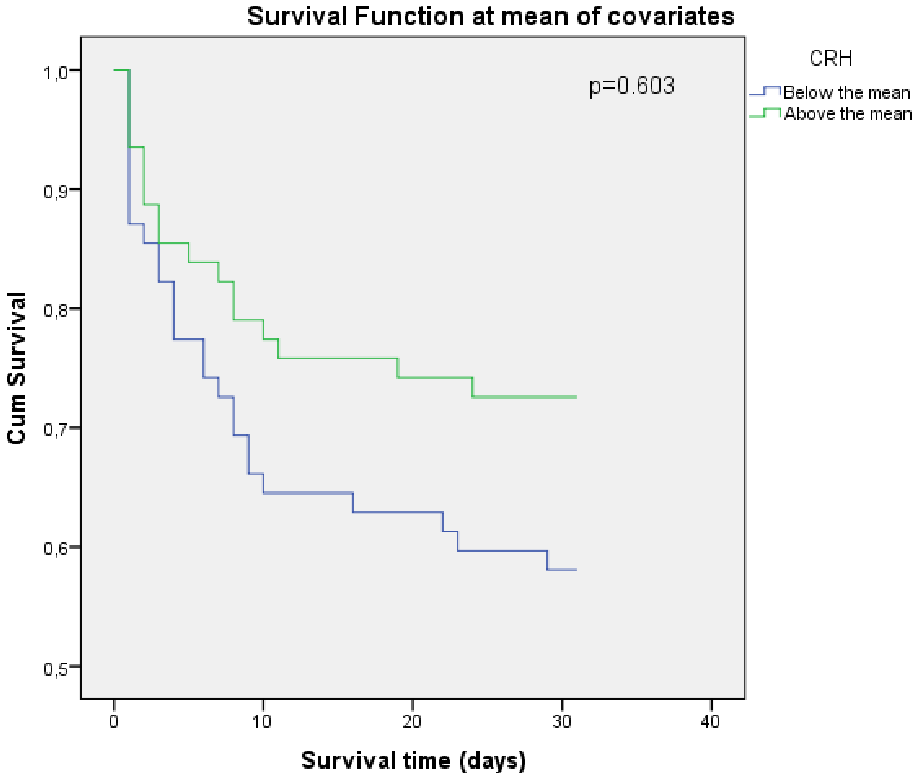

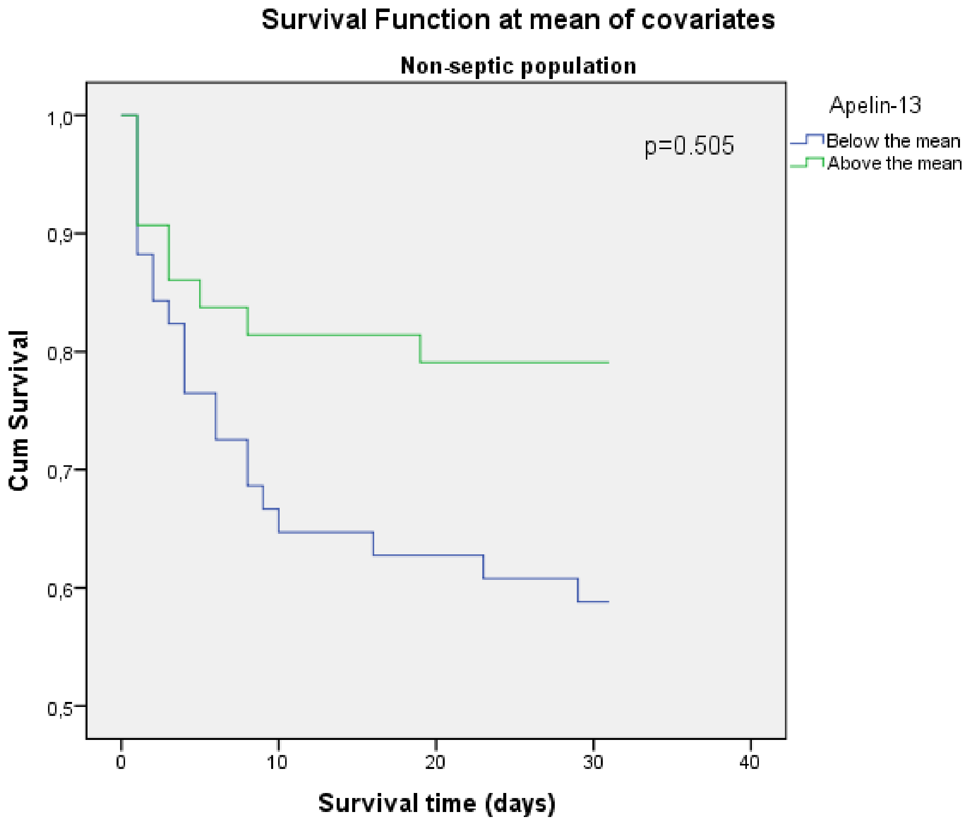
Survival Function at mean of covariates
Non-septic population
Apelin-13
Below the mean
Above the mean
p=0.505
Cum Survival
1,0
0,9
0,8
0,7
0,6
0,5
0
10
20
30
40
Survival time (days)

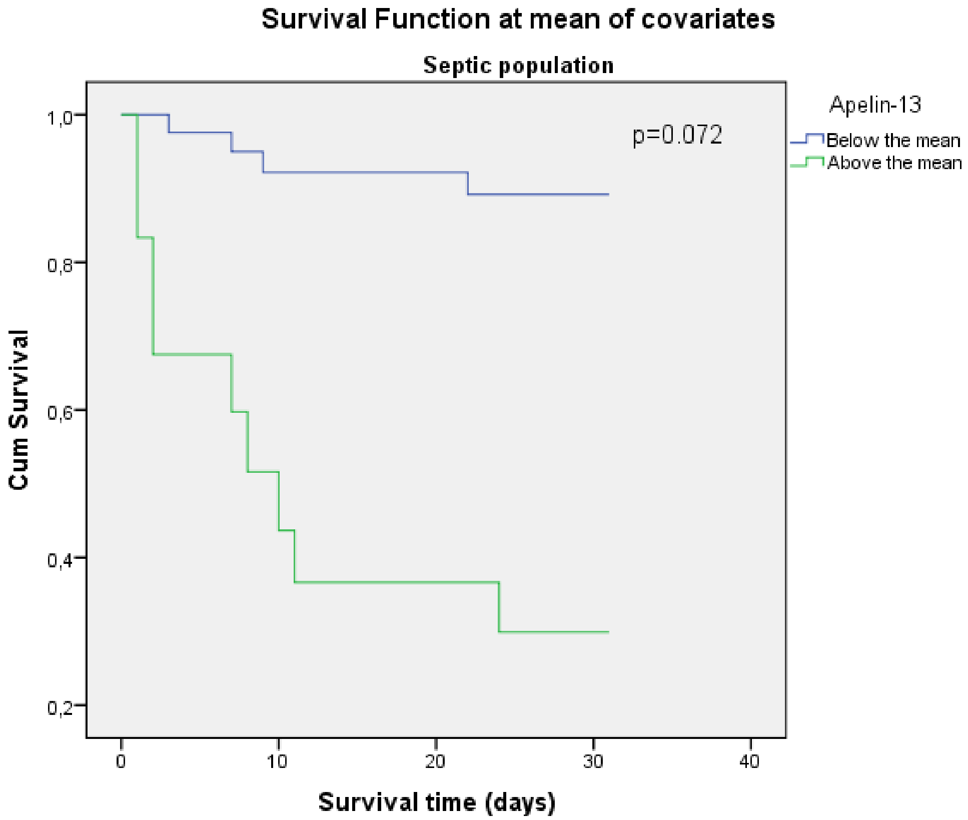
Survival Function at mean of covariates
Septic population
Apelin-13
Below the mean
Above the mean
p=0.072
Cum Survival
1,0
0,8
0,6
0,4
0,2
0
10
20
30
40
Survival time (days)

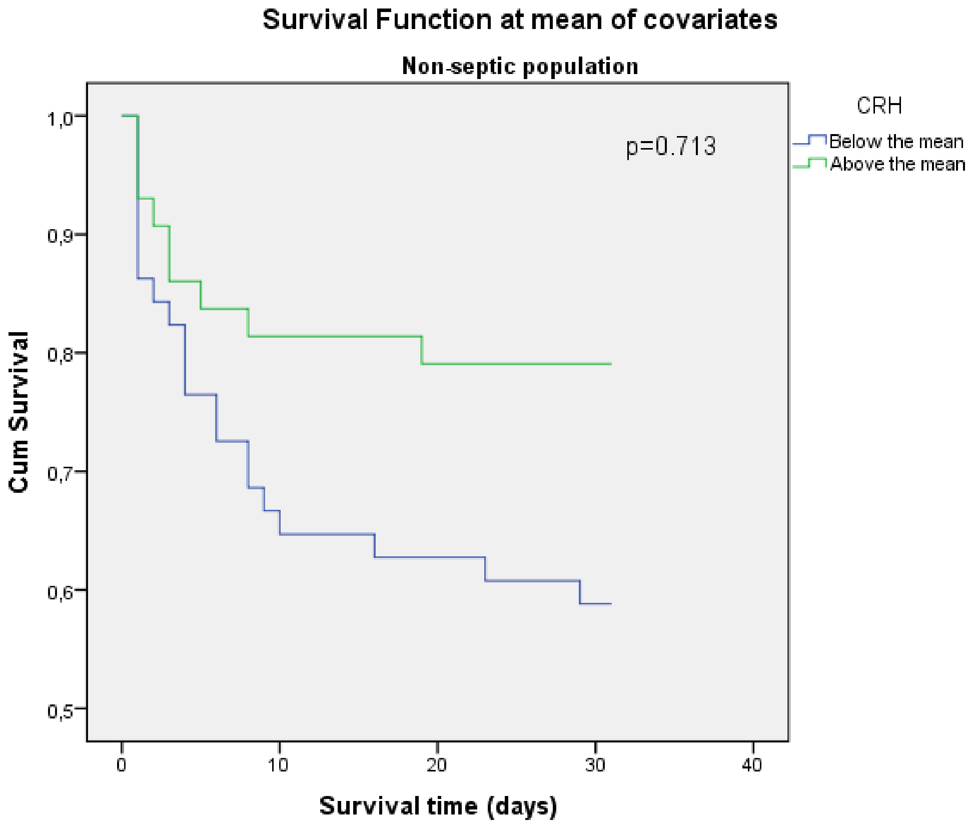
Survival Function at mean of covariates
Non-septic population
CRH
Below the mean
Above the mean
p=0.713
1,0
0,9
0,8
0,7
0,6
0,5
Cum Survival
0
10
20
30
40
Survival time (days)

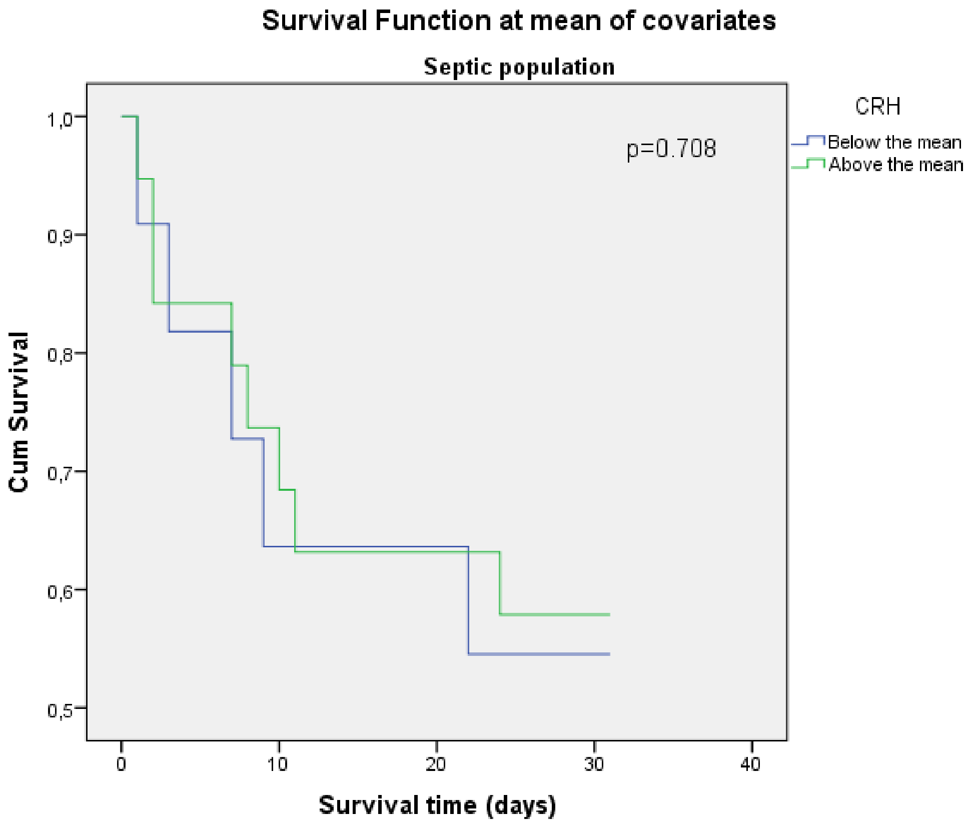
Survival Function at mean of covariates
Septic population
CRH
Below the mean
Above the mean
p=0.708
1,0
0,9
0,8
0,7
0,6
0,5
Cum Survival
0
10
20
30
40
Survival time (days)
